# Risk of subsequent primary melanoma in 68 002 five-year survivors of childhood and adolescent cancer in Europe: the PanCareSurFup cohort study

**DOI:** 10.1093/jnci/djag094

**Published:** 2026-03-30

**Authors:** Behnoush Shojaie, Jop C Teepen, David L Winter, Mike P Gardner, Melanie Kaiser, Desiree Grabow, Cécile M Ronckers, Julianne Byrne, Roderick Skinner, Daniela Alessi, Hama Baqaeen, Rodrigue S Allodji, Helena J H Van Der Pal, Momcilo Jankovic, Francesca Bagnasco, Eva Steliarova-Foucher, Ben D Spycher, Grit Sommer, Edit Bárdi, Thorgerdur Gudmundsdottir, Milena M Maule, Gisela Michel, Monica Muraca, Maria W Gunnes, Line Kenborg, Henrik Hjalgrim, Carlotta Sacerdote, Zsuzsanna Jakab, Riccardo Haupt, Thomas Wiebe, Monica Terenziani, Lorna Zadravec Zaletel, Leontien C M Kremer, Claudia E Kuehni, Päivi M Lähteenmäki, Florent de Vathaire, Lars Hjorth, Michael M Hawkins, Raoul C Reulen

**Affiliations:** Centre for Childhood Cancer Survivor Studies, Department of Applied Health Sciences, University of Birmingham, Birmingham, United Kingdom; Princess Máxima Centre for Paediatric Oncology, Utrecht, The Netherlands; Centre for Childhood Cancer Survivor Studies, Department of Applied Health Sciences, University of Birmingham, Birmingham, United Kingdom; Department of Applied Health Sciences, University of Birmingham, Birmingham, United Kingdom; German Childhood Cancer Registry, Division of Childhood Cancer Epidemiology, Institute of Medical Biostatistics, Epidemiology and Informatics, Johannes-Gutenberg University Mainz, Mainz, Germany; German Childhood Cancer Registry, Division of Childhood Cancer Epidemiology, Institute of Medical Biostatistics, Epidemiology and Informatics, Johannes-Gutenberg University Mainz, Mainz, Germany; German Childhood Cancer Registry, Division of Childhood Cancer Epidemiology, Institute of Medical Biostatistics, Epidemiology and Informatics, Johannes-Gutenberg University Mainz, Mainz, Germany; Division of Childhood and Adolescent and Young Adult (CAYA) Cancer Survivorship Research, German Cancer Research Centre, Heidelberg, Germany; Retired from the Boyne Research Institute, Drogheda, Ireland; Great North Children’s Hospital, Newcastle upon Tyne Hospitals NHS Foundation Trust and Newcastle University Centre for Cancer, Newcastle University, Newcastle upon Tyne, United Kingdom; Childhood Cancer Registry of Piedmont, Cancer Epidemiology Unit, Department of Medical Sciences, University of Turin and CPO-Piemonte, AOU Città della Salute e della Scienza di Torino, Turin, Italy; Centre for Childhood Cancer Survivor Studies, Department of Applied Health Sciences, University of Birmingham, Birmingham, United Kingdom; Radiation Epidemiology Team, Centre for Research in Epidemiology and Population Health, INSERM U1018, University Paris Saclay, Gustave Roussy, Villejuif, France; Radiation Epidemiology (EpiRad) Team, Gustave Roussy, Villejuif, France; Princess Máxima Centre for Paediatric Oncology, Utrecht, The Netherlands; Paediatric Clinic, Maria Letizia Verga Foundation, Instituto di Ricovero e Cura a Carattere Scientifico (IRCCS) San Gerardo, Monza, Italy; Epidemiology and Biostatistics Unit, IRCCS Istituto Giannina Gaslini, Genova, Italy; Cancer Surveillance Branch, International Agency for Research on Cancer, Lyon, France; Childhood Cancer Research Group, Institute of Social and Preventive Medicine, University of Bern, Bern, Switzerland; Childhood Cancer Research Group, Institute of Social and Preventive Medicine, University of Bern, Bern, Switzerland; St Anna Children’s Hospital, Department of Paediatrics and Adolescent Medicine, Vienna, Austria; Department of Paediatrics and Adolescent Medicine, Johannes Kepler University Linz, Kepler University Hospital, Linz, Austria; Children′s Hospital, Landspitali University Hospital, Reykjavik, Iceland; Childhood Cancer Research Group, Danish Cancer Institute, Copenhagen, Denmark; Cancer Epidemiology Unit, Department of Medical Sciences, University of Turin and AOU Citta della Salute e della Scienza, CPO-Piemonte, Turin, Italy; Faculty of Health Sciences and Medicine, University of Lucerne, Lucerne, Switzerland; Diagnosis, Observation, Prevention After Oncologic Treatment (DOPO) clinic, Division of Haematology/Oncology, IRCCS Istituto Giannina Gaslini, Genova, Italy; Department of Registration, Cancer Registry of Norway, Oslo, Norway; National Advisory Unit on Solid Tumours in Childhood, Division of Paediatric and Adolescent Medicine, Oslo University Hospital Rikshospitalet, Oslo, Norway; Childhood Cancer Research Group, Danish Cancer Institute, Copenhagen, Denmark; Haematology, Danish Cancer Institute, Copenhagen, Denmark; Cancer Epidemiology Unit, Department of Medical Sciences, University of Turin and AOU Citta della Salute e della Scienza, CPO-Piemonte, Turin, Italy; Centre for Cancer Epidemiology and Prevention (CPO Piemonte), Turin, Italy; Hungarian Childhood Cancer Registry, Hungarian Paediatric Oncology Network, Department of Paediatrics, Semmelweis University, Budapest, Hungary; Diagnosis, Observation, Prevention After Oncologic Treatment (DOPO) clinic, Division of Haematology/Oncology, IRCCS Istituto Giannina Gaslini, Genova, Italy; Department of Clinical Sciences Lund, Paediatrics, Skane University Hospital, Lund University, Lund, Sweden; Pediatric Oncology Unit, Fondazione IRCCS Istituto Nazionale dei Tumori, Milan, Italy; Division of Radiotherapy, Institute of Oncology, Ljubljana, Slovenia; Faculty of Medicine, University of Ljubljana, Ljubljana, Slovenia; Princess Máxima Centre for Paediatric Oncology, Utrecht, The Netherlands; Emma Children’s Hospital, Department of Pediatrics, Amsterdam Universitair Medisch Centrum (UMC), Amsterdam, The Netherlands; Childhood Cancer Research Group, Institute of Social and Preventive Medicine, University of Bern, Bern, Switzerland; Division of Paediatric Hematology/Oncology, Department of Paediatrics, University Children’s Hospital of Bern, Bern, Switzerland; Department of Paediatrics and Adolescent Medicine, Turku University and Turku University Hospital, Turku, Finland; Radiation Epidemiology Team, Centre for Research in Epidemiology and Population Health, INSERM U1018, University Paris Saclay, Gustave Roussy, Villejuif, France; Radiation Epidemiology (EpiRad) Team, Gustave Roussy, Villejuif, France; Department of Clinical Sciences Lund, Paediatrics, Lund University, Skane University Hospital, Lund, Sweden; Centre for Childhood Cancer Survivor Studies, Department of Applied Health Sciences, University of Birmingham, Birmingham, United Kingdom; Centre for Childhood Cancer Survivor Studies, Department of Applied Health Sciences, University of Birmingham, Birmingham, United Kingdom

## Abstract

**Background:**

Survivors of childhood cancer are at risk of subsequent primary melanoma, but the magnitude of the risk after different childhood cancer types and beyond age 50 years remains inadequately characterized. We quantified risks in the largest cohort of childhood cancer survivors worldwide.

**Methods:**

The PanCare Childhood and Adolescent Cancer Survivor Care and Follow-Up Studies cohort includes 68 002 five-year childhood cancer survivors across Europe (diagnosed 1940-2008). Subsequent primary melanoma risks were quantified using standardized incidence ratios (SIRs), absolute excess risks (AERs), cumulative incidence, and relative risks (RRs).

**Results:**

Over 1 239 675 person-years, 197 subsequent primary melanoma were ascertained, whereas 84.9 were expected (SIR = 2.3, 95% confidence interval [CI] = 2.0 to 2.7). Although the standardized incidence ratio decreased with attained age (*P* for trend < .001), it remained increased at 2.1-fold (95% CI = 1.5 to 3.1) at age 50 years and older. By age 65 years, cumulative incidence was 1.2% (0.8% expected). Of all cancer types, heritable retinoblastoma survivors had the greatest risk (SIR = 16.5, 95% CI = 10.8 to 25.3; AER = 94.4, 95% CI = 59.9 to 148.9), with cumulative incidence of 3.3% at age 50 years and 5.5% at age 60 years. Survivors treated with radiotherapy had a 70% greater risk than those treated without (RR = 1.7, 95% CI = 1.1 to 2.7), increasing to 4.0-fold by age 50 years and older (RR = 3.9, 95% CI = 1.1 to 13.9). For chemotherapy, no statistically significant association was found (RR = 0.9, 95% CI = 0.5 to 1.6).

**Conclusions:**

Childhood cancer survivors, particularly those with heritable retinoblastoma and those treated with radiotherapy, remain at increased risk of melanoma beyond age 50 years. Current survivorship guidelines focus principally on treatment history; however, our findings suggest that long-term follow-up guidelines should additionally include childhood cancer type and attained age as risk-stratifying factors, particularly where individual treatment records are unavailable.

## Introduction

Since the 1970s, survival from childhood cancer has improved substantially, with 5-year survival rates currently reaching 81% in Europe.^1^ Advancements in cancer treatment have led to increases in survival; however, the long-term effects of treatment remain a major concern with two-thirds of survivors experiencing 1 or more late effects.^2^^,^^3^ One of the most severe late effects is the development of a subsequent primary neoplasm with 14% of survivors experiencing a subsequent primary neoplasm by age 60 years.^4^ A diagnosis of melanoma is especially concerning if metastasized to distant organs because of its high mortality rate.^5-8^ As the population of childhood cancer survivors reaches age 50 years and beyond—approaching the median age of melanoma diagnosis in the general population (age 59 years)^9^—even a modestly increased risk of melanoma relative to the general population could result in a substantial absolute excess risk among survivors.

Previous large-scale cohort studies have quantified the risks of subsequent primary melanomas among survivors of childhood cancer,^4^^,^^10-14^ but to our knowledge, the largest study to date that has characterized the risk of melanoma by specific factors included 110 invasive melanomas but only 10 melanomas that occurred beyond age 50 years.^11^ In addition, the magnitude of the risk of subsequent primary melanomas among specific types of childhood cancer remains poorly characterized. Hence, we conducted a retrospective cohort study involving nearly 68 000 five-year survivors of childhood cancer with nearly 3 times the number of melanomas beyond age 50 years. The principal aim of this study was to quantify the long-term risks of developing subsequent primary melanomas by specific factors, including childhood cancer type, sex, age at childhood cancer, treatment era, attained age, and previous treatment with radiotherapy and chemotherapy, using the largest cohort of childhood cancer survivors worldwide.

## Methods

### PanCare Childhood and Adolescent Cancer Survivor Care and Follow-Up Studies

PanCare is a multidisciplinary pan-European network of professionals, researchers, childhood cancer survivors, and their families. One of the main research projects of PanCare was the PanCare Childhood and Adolescent Cancer Survivor Care and Follow-Up Studies (PanCareSurFup). One of the principal aims of the PanCareSurFup was to investigate the risks and causes of developing subsequent primary neoplasms among 5-year survivors of childhood and adolescent cancer.^15-17^ The study includes survivors diagnosed with cancer at age 0-20 years between 1940 and 2008, representing the largest assembled cohort of childhood cancer survivors to date. Data on individuals were collected from 13 institutions across 12 European countries including France, Hungary, Italy, the Netherlands, Denmark, Finland, Iceland, Norway, Sweden, Slovenia, Switzerland, and the United Kingdom (Table S1). The data were obtained from 11 population-based cancer registries and 2 major hospital treatment centers. Ethical approval had already been obtained for each institution contributing to the PanCareSurFup cohort, and each institution obtained either written informed consent from participants or the necessary legal permission to process individual patient data without consent.

### Cohort ascertainment and childhood cancer classification

To ensure standardized classification across countries, all childhood cancers were coded according to the *International Classification of Diseases for Oncology, Third Edition* (*ICD-O-3*). For diagnoses originally coded in earlier *ICD-O* versions, topography and morphology codes were converted using the International Association of Cancer Registries (IARC)/IACR Check and Conversion Program.^18^ Following this conversion, childhood cancers were then categorized into major diagnostic groups according to the *International Classification of Childhood Cancer, Third Edition* (*ICCC-3*).^19^ Only malignant childhood tumors were included in the cohort except for intracranial benign tumors and bladder tumors. To minimize potential misclassification of a recurrence of a first primary melanoma as a subsequent primary melanoma, we excluded individuals with childhood melanoma. We also excluded individuals with a primary diagnosis of myelodysplastic syndrome, Langerhans cell histiocytosis, or chronic myeloproliferative and lymphoproliferative disorders because of inconsistent ascertainment of these conditions across countries.

### Subsequent primary melanoma ascertainment

Subsequent primary melanomas were ascertained through linkage with multiple complementary sources including population-based national cancer registries, medical records and hospital data, national mortality records, health insurance registries, late effect clinics, and questionnaires. All subsequent primary melanomas were validated through pathology reports. We included only cutaneous malignant melanomas (*ICD-10* code C43) if the behavior code was malignant (*ICD-O* behavior code: 3). Ocular melanomas and other noncutaneous sites were excluded as they are etiologically distinct from cutaneous melanoma and there were too few (*n* = 8) for meaningful analyses.

### General population melanoma rates

For each country, general population melanoma incidence rates were obtained from the Cancer Incidence in Five Continents database.^20^ For Hungary, where national data were unavailable, we used mortality-adjusted melanoma incidence rates from Slovakia.^21^ Where incidence rates for specific calendar years were missing, rates from the closest available year were used.

### Statistical analysis

Time-at-risk for subsequent primary melanomas began 5 years after childhood cancer diagnosis and continued until loss to follow-up, death, or study exit date. Standardized incidence ratios (SIRs) were calculated as SIR = ∑Observed/∑Expected number of melanoma.^22^ Expected numbers were derived by accumulating person-years at risk within specific strata of the factors country, sex, age (5-year bands), and calendar year (1-year bands) and then multiplying the aggregated person-years within each stratum by the corresponding stratum-specific general population melanoma incidence rate. Absolute excess risks (AERs) were calculated as AER = [(∑Observed - ∑Expected number of melanoma)/∑Person-years at risk] × 100 000. The absolute excess risk is an additive measure, and in this study, it quantifies the additional number of melanomas diagnosed among survivors on top of those diagnosed in the general population, per 100 000 person-years. All melanomas, including multiple primaries in the same individual, were included in standardized incidence ratio and absolute excess risk calculations to ensure appropriate comparability with general population rates.

To account for potential confounding, we used random effects multivariable Poisson regression—with the expected number of melanomas as offset—to calculate relative risks (RRs) of melanoma by various potential explanatory factors including sex, type of childhood cancer, hereditary status for retinoblastoma survivors, age at diagnosis, decade of diagnosis, attained age, treatment with radiotherapy (yes, no), and chemotherapy (yes, no). In this context, the relative risk can be interpreted as a ratio of standardized incidence ratios adjusted for confounders, with the lowest level of each factor acting as the reference category. For each potential explanatory factor, we fitted a separate Poisson regression model with confounder selection informed by a directed acyclic graph. This approach ensured adjustment for a theoretically justified set of confounding variables based on causal assumptions depicted in the directed acyclic graph. Country was included as a random effect in the Poisson regression model to account for potential between-country heterogeneity arising from unmeasured variation in, for example, genetic background, health-care systems, diagnostic practices, and treatment protocols.

The cumulative incidence of a first subsequent primary melanoma was calculated by attained age, accounting for death as a competing risk.^23^^,^^24^ The expected cumulative incidence was estimated by the conditional method using general population melanoma incidence rates.^25^

Likelihood-ratio tests were used to calculate *P* values for heterogeneity and linear trend. Variables were coded as consecutive integer values (eg, 1, 2, 3, 4 for attained age) and included in the models as factors for heterogeneity tests and as continuous variables for trend tests. A 2-sided statistical significance level was set to less than 5%. All analyses were conducted using Stata version 18.0.

## Results

### Cohort characteristics

The cohort comprised 68 002 individuals followed-up for 1 239 675 person-years, with a median follow-up of 16.3 years (IQR = 6.9-27.3 years). Loss to follow-up did not exceed 6% in any country (Table S1). Among 188 survivors, 197 subsequent primary melanomas were observed, with 9 survivors developing more than 1 melanoma. The median attained age at cohort exit was 30.2 years (IQR = 21.0-40.4 years). The median latency to subsequent primary melanoma development was 26.5 years (IQR = 19.2-36.5 years) (Table S2). The most common anatomical sites were the trunk (36.0%) and lower limb (27.9%). The most frequent type of melanoma was superficial spreading melanoma (50.3%) (Table 1).

**Table 1.** Cohort characteristics and subsequent primary melanoma ascertainment among 68 002 five-year childhood cancer survivors in the PanCareSurFup study.

Characteristics	Entire cohort, No. (%)^a^	Subsequent primary melanoma, No. (%)^a^
All survivors	68 002 (100)	197 (100)
Sex		
Male	37 182 (54.3)	81 (41.1)
Female	30 820 (45.7)	116 (58.9)
Childhood cancer type		
Leukemia	16 646 (24.5)	29 (14.7)
Hodgkin lymphoma	6046 (8.9)	17 (8.6)
Non-Hodgkin lymphoma	4078 (6.0)	12 (6.1)
Central nervous system tumor	14 592 (21.5)	27 (13.7)
Neuroblastoma	3178 (4.7)	8 (4.1)
Retinoblastoma	2590 (3.8)	36 (18.3)
Hereditary	714 (1.0)	21 (10.7)
Nonhereditary	1013 (1.5)	7 (3.6)
Unknown	863 (1.3)	8 (4.1)
Wilms tumor	4783 (7.0)	13 (6.6)
Osteosarcoma	3173 (4.7)	5 (2.5)
Soft-tissue sarcoma	4531 (6.7)	17 (8.6)
Germ cell and gonadal neoplasm	2721 (4.0)	11 (5.6)
Other^b^	5664 (8.3)	22 (11.2)
Data provider country		
France	3130 (4.6)	8 (4.1)
Hungary	4869 (7.2)	1 (0.5)
Italy	8815 (13.0)	6 (3.0)
Netherlands	6022 (8.9)	22 (11.2)
Denmark	4615 (6.8)	19 (9.6)
Sweden	7418 (10.9)	13 (6.6)
Norway	3537 (5.2)	9 (4.6)
Finland	6056 (8.9)	19 (9.6)
Iceland	261 (0.4)	0
Slovenia	1221 (1.8)	0
Switzerland	4239 (6.2)	4 (2.0)
United Kingdom	17 819 (26.2)	96 (48.7)
Age at prior treatment, y		
0-4	26 603 (39.1)	84 (42.6)
5-9	15 611 (23.0)	39 (19.8)
10-14	15 196 (22.3)	49 (24.9)
15-19	10 592 (15.6)	25 (12.7)
Decade of childhood cancer diagnosis		
Before 1970	8715 (12.8)	70 (35.5)
1970-1979	13 246 (19.5)	64 (32.5)
1980-1989	20 506 (30.2)	45 (22.8)
1990-1999	18 843 (27.7)	17 (8.6)
2000-2008	6692 (9.8)	1 (0.5)
Attained age, y		
5-19	15 352 (22.6)	21 (10.7)
20-29	18 424 (27.1)	49 (24.9)
30-39	16 707 (24.6)	59 (29.9)
40-49	10 720 (15.8)	39 (19.8)
50 and older	6799 (9.9)	29 (14.7)
Body site (*ICD-10* code classification)		
Above neck^c^	N/A	24 (12.2)
Trunk (C43.5)	N/A	71 (36.0)
Upper limb (C43.6)	N/A	37 (18.8)
Lower limb (C43.7)	N/A	55 (27.9)
Overlapping (C43.8)	N/A	1 (0.5)
Type of malignancy (*ICD*-O-3 code classification)		
Malignant melanoma, not otherwise specified (8720/3)	N/A	78 (39.6)
Nodular melanoma (8721/3)	N/A	13 (6.6)
Amelanotic melanoma (8730/3)	N/A	1 (0.5)
Lentigo maligna melanoma (8742/3)	N/A	3 (1.5)
Superficial spreading melanoma (8743/3)	N/A	99 (50.3)
Malignant melanoma in giant pigmented nevus (8761/3)	N/A	2 (1.0)
Initial radiotherapy^d^		
Yes	21 106 (57.1)	89 (71.8)
No	15 852 (42.9)	35 (28.2)
Initial chemotherapy^d^		
Yes	30 947 (74.5)	62 (54.4)
No	10 612 (25.5)	52 (45.6)

Abbreviations: *ICD* = *International Classification of Diseases for Oncology*; N/A = not applicable; PanCareSurFup = PanCare Childhood and Adolescent Cancer Survivor Care and Follow-Up Studies.

a Counts for the PanCareSurFup cohort are based on the total number of individual survivors. Counts for subsequent primary melanoma are based on the total number of subsequent primary melanoma observations.

b Included nonmelanoma skin cancers, thyroid carcinomas, other and unspecified carcinomas, unspecified lymphoma, nasopharyngeal carcinoma, renal carcinomas, other peripheral nervous cell tumors, other and unspecified malignant neoplasms.

c Included eye (C43.1), ear (C43.2), other part of face (C43.3), and scalp and neck (C43.4).

d A total of 21 887 individuals from Nordic countries (Denmark, Sweden, Norway, Finland, Iceland) were excluded because of lack of treatment data.

### Risk by type of childhood cancer

Overall, survivors were 2.3 times more likely to develop a subsequent primary melanoma than expected (SIR = 2.3, 95% confidence interval [CI] = 2.0 to 2.7), with 9 melanomas per 100 000 person-years in excess of that expected in the general population (AER = 9.0, 95% CI = 7.1 to 11.6). In terms of the standardized incidence ratio, survivors of heritable retinoblastoma were at greatest risk (SIR = 16.5, 95% CI = 10.8 to 25.3) followed by nonheritable retinoblastoma (SIR = 3.2, 95% CI = 1.5 to 6.7), neuroblastoma (SIR = 2.8, 95% CI = 1.4 to 5.6), germ cell and gonadal neoplasm (SIR = 2.6, 95% CI = 1.4 to 4.7), and non-Hodgkin lymphoma (SIR = 2.5, 95% CI = 1.4 to 4.4) survivors. Other childhood cancers, except osteosarcoma, showed elevated risks although of lesser magnitude (Table 2). The greatest absolute excess risks were found among survivors of heritable retinoblastoma (AER = 94.4, 95% CI = 59.9 to 148.9) and nonheritable retinoblastoma (AER = 15.3, 95% CI = 5.2 to 45.1). Multivariable analyses revealed that heritable retinoblastoma survivors were at 5.2-fold greater risk than nonheritable retinoblastoma survivors (95% CI = 2.2 to 12.2).

**Table 2.** Standardized incidence ratio, absolute excess risk, and relative risk of subsequent primary melanoma in all survivors and by whether treated with or without radiotherapy.

Factor	All survivors				With radiotherapy^a^			Without radiotherapy		
	O/E	SIR (95% CI)	AER (95% CI)	RR^b^ (95% CI)	O/E	SIR (95% CI)	AER (95% CI)	O/E	SIR (95% CI)	AER (95% CI)
Overall	197/84.9	2.3 (2.0 to 2.7)	9.0 (7.1 to 11.6)		89/34.9	2.6 (2.1 to 3.1)	11.1 (7.9 to 15.6)	35/18.2	1.9 (1.4 to 2.7)	6.0 (3.0 to 11.9)
Sex^b1^										
Male	81/35.6	2.3 (1.8 to 2.8)	6.8 (4.6 to 10.0)	1.0 (referent)	38/15.0	2.5 (1.8 to 3.5)	8.6 (5.1 to 14.6)	12/7.4	1.6 (0.9 to 2.9)	3.1 (0.7 to 13.4)
Female	116/49.3	2.4 (2.0 to 2.8)	11.6 (8.5 to 16.0)	1.0 (0.8 to 1.4)	51/19.8	2.6 (2.0 to 3.4)	14.1 (9.0 to 22.0)	23/10.8	2.1 (1.4 to 3.2)	9.3 (4.3 to 20.1)
*P* for heterogeneity		.81	.03	.81		.94	.16		.45	.14
Childhood cancer types										
Leukemia	29/13.7	2.1 (1.5 to 3.0)	5.9 (3.0 to 11.8)		19/9.4	2.0 (1.3 to 3.2)	6.3 (2.6 to 15.4)	4/2.3	1.8 (0.7 to 4.7)	3.5 (0.4 to 33.9)
Hodgkin lymphoma	17/8.0	2.1 (1.3 to 3.4)	9.1 (3.7 to 22.4)		7/3.8	1.8 (0.9 to 3.8)	6.7 (1.3 to 34.5)	1/0.8	1.2 (0.2 to 8.5)	1.6 (0.0 to *)
NHL^c^	12/4.9	2.5 (1.4 to 4.4)	10.2 (3.9 to 26.3)		5/2.1	2.4 (1.0 to 5.8)	11.4 (2.5 to 51.1)	1/1.1	0.9 (0.1 to 6.5)	0.0
CNS tumor	27/19.2	1.4 (1.0 to 2.1)	3.0 (0.8 to 10.9)		9/6.9	1.3 (0.7 to 2.5)	2.2 (0.1 to 37.8)	6/4.2	1.4 (0.6 to 3.2)	3.4 (0.2 to 46.2)
Neuroblastoma	8/2.9	2.8 (1.4 to 5.6)	8.3 (2.8 to 24.4)		2/1.2	1.6 (0.4 to 6.4)	3.6 (0.1 to 143.3)	4/1.0	4.0 (1.5 to 10.8)	12.7 (3.5 to 46.8)
Retinoblastoma	36/4.4	8.3 (6.0 to 11.5)	44.8 (30.9 to 65.0)		20/1.4	13.8 (8.9 to 21.5)	82.1 (51.2 to 131.7)	5/1.5	3.4 (1.4 to 8.1)	17.7 (5.1 to 61.7)
Wilms tumor	13/6.0	2.2 (1.3 to 3.7)	6.4 (2.3 to 17.6)		10/3.7	2.7 (1.5 to 5.0)	11.5 (4.3 to 30.7)	2/1.0	2.1 (0.5 to 8.3)	4.6 (0.3 to 67.1)
Osteosarcoma	5/5.2	1.0 (0.4 to 2.3)	0.0		1/1.8	0.6 (0.1 to 4.0)	0.0	3/1.4	2.2 (0.7 to 6.7)	10.2 (1.3 to 82.8)
Ewing sarcoma	0/1.3	0.0	0.0		0	0.0	0.0	0	0.0	0.0
Soft-tissue sarcoma	17/6.9	2.4 (1.5 to 3.9)	10.8 (4.8 to 24.1)		8/2.3	3.5 (1.8 to 7.0)	18.9 (7.2 to 49.7)	1/1.9	0.5 (0.1 to 3.7)	0.0
Germ cell and gonadal neoplasm^d^	11/4.2	2.6 (1.4 to 4.7)	13.8 (5.3 to 36.2)		1/0.7	1.5 (0.2 to 10.6)	5.1 (0.0 to 2027.1)	4/1.2	3.4 (1.3 to 9.1)	19.1 (4.8 to 76.3)
Other^e^	22/9.5	2.3 (1.5 to 3.5)	11.8 (5.7 to 24.5)		7/1.5	4.5 (2.2 to 9.5)	32.3 (12.5 to 83.5)	4/1.9	2.1 (0.8 to 5.6)	8.6 (1.3 to 55.1)
*P* for heterogeneity		<.001	<.001			<.001	<.001		.57	.65
Retinoblastoma, hereditary status^b2^										
Nonheritable	7/2.2	3.2 (1.5 to 6.7)	15.3 (5.2 to 45.1)	1.0 (referent)	2/0.4	5.6 (1.4 to 22.3)	31.1 (5.7 to 168.3)	3/1.3	2.3 (0.7 to 7.1)	10.2 (1.4 to 75.7)
Heritable	21/1.3	16.5 (10.8 to 25.3)	94.4 (59.9 to 148.9)	5.2 (2.2 to 12.2)	18/1.1	16.7 (10.5 to 26.5)	98.4 (60.2 to 160.9)	2/0.1	13.6 (3.4 to 54.4)	70.9 (15.9 to 316.3)
*P* for heterogeneity		<.001	<.001	<.001		.21	.26		.19	.27
Treatment era^b3^										
Before 1970	70/30.0	2.3 (1.8 to 3.0)	13.3 (8.8 to 20.1)	1.0 (referent)	26/10.2	2.5 (1.7 to 3.7)	14.3 (7.6 to 26.9)	10/5.5	1.8 (1.0 to 3.4)	8.4 (2.1 to 33.2)
1970-1979	64/25.9	2.5 (1.9 to 3.2)	11.0 (7.3 to 16.5)	1.1 (0.8 to 1.5)	37/13.6	2.7 (2.0 to 3.8)	13.2 (7.9 to 21.9)	13/5.1	2.6 (1.5 to 4.4)	12.2 (5.0 to 29.8)
1980-1989	45/22.5	2.0 (1.5 to 2.7)	5.7 (3.2 to 10.3)	0.9 (0.6 to 1.3)	20/9.3	2.1 (1.4 to 3.3)	6.9 (3.0 to 15.6)	10/5.7	1.7 (0.9 to 3.2)	4.5 (1.1 to 19.3)
1990-1999	17/6.1	2.8 (1.7 to 4.5)	5.8 (3.0 to 13.1)	1.2 (0.7 to 2.1)	6/1.6	3.7 (1.7 to 8.3)	10.8 (3.6 to 32.2)	2/1.7	1.2 (0.3 to 4.6)	0.5 (0.0 to 10 425.1)
2000-2008	1/0.4	2.4 (0.3 to 16.8)	2.4 (0.1 to 71.8)	1.0 (0.1 to 7.7)	0/0.1	0.0	0.0	0/0.2	0.0	0.0
*P* for trend		.89	.01	.70		.95	.18		.52	.08
Age at prior treatment,^b4^ y										
0-4	84/24.7	3.4 (2.7 to 4.2)	11.3 (8.4 to 15.3)	1.0 (referent)	49/12.3	4.0 (3.0 to 5.3)	16.7 (11.5 to 24.2)	13/6.3	2.1 (1.2 to 3.6)	4.9 (1.7 to 13.9)
5-9	39/18.7	2.1 (1.5 to 2.9)	7.0 (3.8 to 12.8)	0.8 (0.6 to 1.3)	17/9.8	1.7 (1.1 to 2.8)	5.2 (1.7 to 16.0)	10/4.2	2.4 (1.3 to 4.5)	9.2 (3.2 to 26.6)
10-14	49/25.9	1.9 (1.4 to 2.5)	8.2 (4.5 to 14.8)	0.7 (0.5 to 1.2)	23/11.3	2.0 (1.3 to 3.1)	9.9 (4.4 to 22.1)	10/6.6	1.5 (0.8 to 2.8)	4.9 (0.8 to 30.9)
15-21	25/15.6	1.6 (1.1 to 2.4)	6.6 (2.3 to 18.6)	0.6 (0.3 to 1.0)	0/1.4	0	0	2/1.1	1.8 (0.4 to 7.2)	8.6 (0.4 to 195.8)
*P* for trend		<.001	.13	.24		<.001	.02		.49	.77
Attained age,^b5^ y										
Younger than 20	21/3.0	7.0 (4.6 to 10.7)	4.4 (2.7 to 7.3)	1.0 (referent)	9/1.0	8.7 (4.5 to 16.8)	5.3 (2.5 to 11.1)	3/0.9	3.3 (1.1 to 10.3)	1.9 (0.4 to 9.5)
20-29	49/20.5	2.4 (1.8 to 3.2)	7.0 (4.3 to 11.3)	0.4 (0.2 to 0.6)	25/7.5	3.3 (2.3 to 4.9)	11.2 (6.4 to 19.6)	5/4.6	1.1 (0.5 to 2.6)	0.5 (0.0 to *)
30-39	59/27.9	2.1 (1.6 to 2.7)	12.2 (7.5 to 19.8)	0.3 (0.2 to 0.5)	22/12.1	1.8 (1.2 to 2.8)	9.1 (3.6 to 23.0)	17/5.5	3.1 (1.9 to 4.9)	23.5 (11.6 to 47.6)
40-49	39/19.9	2.0 (1.4 to 2.7)	16.4 (8.6 to 31.0)	0.3 (0.1 to 0.5)	17/8.8	1.9 (1.2 to 3.1)	15.7 (5.9 to 42.0)	7/4.1	1.7 (0.8 to 3.6)	12.4 (2.1 to 72.4)
50 and older	29/13.7	2.1 (1.5 to 3.1)	30.6 (15.4 to 60.9)	0.3 (0.1 to 0.6)	16/5.5	2.9 (1.8 to 4.8)	51.4 (24.4 to 108.4)	3/3.1	1.0 (0.3 to 3.0)	0
*P* for trend		<.001	<.001	<.001		.02	<.001		.37	.05
Chemotherapy^b6^										
No	52/23.1	2.2 (1.7 to 3.0)	10.5 (6.4 to 17.1)	1.0 (referent)	34/13.2	2.6 (1.8 to 3.6)	13.7 (7.9 to 23.8)	18/9.8	1.8 (1.2 to 2.9)	6.8 (2.5 to 18.7)
Yes	62/29.5	2.1 (1.6 to 2.7)	6.2 (3.9 to 10.0)	0.9 (0.5 to 1.6)	43/19.6	2.2 (1.6 to 3.0)	7.5 (4.3 to 13.0)	13/7.6	1.7 (1.0 to 2.9)	3.6 (1.0 to 13.5)
*P* for heterogeneity		.72	.15	.73		.49	.14		.83	.45
Radiotherapy^b6^										
No	35/18.2	1.9 (1.4 to 2.7)	6.0 (3.0 to 11.9)	1.0 (referent)						
Yes	89/34.9	2.6 (2.1 to 3.1)	11.1 (7.9 to 15.6)	1.7 (1.1 to 2.7)						
*P* for heterogeneity		.15	.08	.02						

Abbreviations: AER = absolute excess risk; CI = confidence interval; CNS = central nervous system; E = expected number; NHL = non-Hodgkins lymphoma; O = observed number; RR = relative risk; SIR = standardized incidence ratio.

a It is notable that treatment data were available for 68% of the total cohort.

b Relative risks were derived from 6 different Poisson regression models. For each exposure (factor) a specific Poisson regression model was applied including a different set of confounders. A directed acyclic graph was designed to guide the potential confounding factors. Country was considered as a random effect in each model. b1: No adjustment required (see https://dagitty.net/mHtRRBr55). b2: No adjustment required (see https://dagitty.net/mGy2bhQAf). b3: No adjustment required (See: https://dagitty.net/m8u8W5oFt). b4: Relative risks were derived from a Poisson regression including age at diagnosis and type of childhood cancer (see https://dagitty.net/mVAmNzxtn). b5: Relative risks were derived from a Poisson regression including attained age, type of childhood cancer, decade of childhood cancer, and age at diagnosis (see https://dagitty.net/mRGomGpWk). b6: Relative risks were derived from a Poisson regression including chemotherapy, radiotherapy, type of childhood cancer, and decade of childhood cancer (see https://dagitty.net/mGghodMWs).

c Includes 10 NHL and 2 Burkitt lymphoma.

d Excluded CNS germ cells tumors and added to CNS tumors category.

e Included 11 observed melanomas in nonmelanoma skin cancer, 2 in thyroid carcinomas, 3 in other and unspecified carcinomas, 2 in unspecified lymphomas, 1 in nasopharyngeal carcinoma, 1 in renal carcinoma, 1 in other peripheral nervous cell tumor, 1 in other and unspecified malignant neoplasm.

### Risk by attained age

The standardized incidence ratio decreased with increasing attained age (*P* for trend < .001) but was still 2.0-fold elevated (95% CI = 1.4 to 2.7) at ages 40-49 years and 2.1-fold (95% CI = 1.5 to 3.1) beyond age 50 years. Multivariable analyses confirmed this pattern, showing a 70% decrease in the relative risk from ages 5-19 years to 50 years and older (Table 2). Even after excluding retinoblastoma survivors, this trend persisted (*P* for trend < .001) with individuals aged 40-49 years and 50 years and older at 60% (SIR = 1.6, 95% CI = 1.1 to 2.3) and 80% (SIR = 1.8, 95% CI = 1.2 to 2.7) elevated risk, respectively (Table 3). Conversely, the overall absolute excess risk increased with attained age from 4.4 (95% CI = 2.7 to 7.3) at ages 5-19 years to 16.4 (95% CI = 8.6 to 31.0) at ages 40-49 and 30.6 (95% CI = 15.4 to 60.9) at age 50 years and older (*P* trend < .001) (Table 2). This trend also persisted after excluding retinoblastoma survivors (*P* trend < .001) (Table 3).

**Table 3.** Standardized incidence ratio, absolute excess risk, and relative risk of subsequent primary melanoma without retinoblastoma survivors.

Factor	All survivors				With radiotherapy^a^			Without radiotherapy		
	O/E	SIR (95% CI)	AER (95% CI)	RR^b^ (95% CI)	O/E	SIR (95% CI)	AER (95% CI)	O/E	SIR (95% CI)	AER (95% CI)
Overall	161/80.5	2.0 (1.7 to 2.3)	6.9 (5.1 to 9.4)		69/33.4	2.1 (1.6 to 2.6)	7.6 (4.8 to 12.1)	30/16.7	1.8 (1.3 to 2.6)	5.1 (2.3 to 11.4)
Sex^b1^										
Male	63/33.8	1.9 (1.5 to 2.4)	4.6 (2.7 to 7.9)	1.0 (referent)	29/14.4	2.0 (1.4 to 2.9)	5.7 (2.8 to 11.8)	9/6.8	1.3 (0.7 to 2.5)	1.6 (0.1 to 22.9)
Female	98/46.7	2.1 (1.7 to 2.6)	9.5 (6.5 to 13.9)	1.1 (0.8 to 1.6)	40/19.0	2.1 (1.5 to 2.9)	9.9 (5.5 to 17.9)	21/9.9	2.1 (1.4 to 3.3)	9.2 (4.1 to 20.7)
*P* for heterogeneity		.46	.03	.45		.87	.24		.22	.07
Treatment era^b2^										
Before 1970	48/27.1	1.8 (1.3 to 2.3)	7.8 (4.1 to 15.0)	1.0 (referent)	16/9.2	1.7 (1.1 to 2.8)	6.9 (2.2 to 21.9)	8/4.5	1.8 (0.9 to 3.6)	8.1 (1.7 to 39.4)
1970-1979	53/24.9	2.1 (1.6 to 2.8)	8.5 (5.1 to 14.2)	1.1 (0.7 to 1.8)	30/13.3	2.3 (1.6 to 3.2)	9.7 (5.1 to 18.4)	10/4.7	2.1 (1.1 to 3.9)	8.8 (2.7 to 28.6)
1980-1989	43/22.0	2.0 (1.4 to 2.6)	5.5 (3.0 to 10.2)	0.9 (0.5 to 1.4)	18/9.2	2.0 (1.2 to 3.1)	5.8 (2.3 to 15.0)	10/5.6	1.8 (1.0 to 3.3)	4.8 (1.2 to 19.7)
1990- 1999	17/6.5	2.6 (1.6 to 4.2)	5.4 (2.5 to 11.7)	1.1 (0.5 to 2.1)	5/1.7	2.9 (1.2 to 7.1)	7.5 (2.0 to 28.2)	2/1.9	1.1 (0.3 to 4.2)	0.2 (0.0 to 1.1e + 10)
*P* for trend		.27	.29	.85		.48	.76		.60	.14
Age at prior treatment,^b3^ y										
0-4	50/20.7	2.4 (1.8 to 3.2)	6.4 (4.0 to 10.3)	1.0 (referent)	29/10.9	2.7 (1.8 to 3.8)	9.1 (5.1 to 16.4)	9/4.9	1.8 (1.0 to 3.5)	3.4 (0.8 to 14.4)
5-9	37/18.4	2.0 (1.5 to 2.8)	6.5 (3.4 to 12.3)	0.9 (0.6 to 1.4)	17/9.8	1.7 (1.1 to 2.8)	5.2 (1.7 to 16.0)	9/4.0	2.2 (1.2 to 4.3)	8.0 (2.4 to 26.1)
10-14	49/25.9	1.9 (1.4 to 2.5)	8.2 (4.5 to 14.8)	0.9 (0.5 to 1.4)	23/11.3	2.0 (1.3 to 3.1)	9.9 (4.4 to 22.1)	10/6.6	1.5 (0.8 to 2.8)	4.9 (0.8 to 30.9)
15-21	25/15.6	1.6 (1.1 to 2.4)	6.6 (2.3 to 18.6)	0.7 (0.4 to 1.2)	0/1.4	0	0	2/1.1	1.8 (0.4 to 7.2)	8.6 (0.4 to 195.8)
*P* for trend		.08	.70	.21		.09	.52		.69	.59
Attained age,^b4^ y										
Younger than 20	18/2.8	6.3 (4.0 to 10.1)	4.0 (2.3 to 6.9)	1.0 (referent)	6/1.0	6.1 (2.7 to 13.5)	3.5 (1.4 to 9.2)	3/0.9	3.5 (1.1 to 10.8)	2.1 (0.4 to 10.1)
20-29	42/19.6	2.1 (1.6 to 2.9)	5.7 (3.2 to 10.1)	0.3 (0.2 to 0.6)	21/7.2	2.9 (1.9 to 4.4)	9.1 (4.7 to 17.5)	5/4.4	1.1 (0.5 to 2.7)	0.8 (0.0 to 855.2)
30-39	48/26.6	1.8 (1.4 to 2.4)	8.8 (4.7 to 16.6)	0.3 (0.2 to 0.5)	18/11.7	1.5 (1.0 to 2.4)	6.0 (1.6 to 22.5)	14/5.2	2.7 (1.6 to 4.6)	19.6 (8.5 to 44.9)
40-49	30/18.7	1.6 (1.1 to 2.3)	10.3 (4.0 to 26.6)	0.3 (0.1 to 0.5)	14/8.4	1.7 (1.0 to 2.8)	11.3 (3.1 to 41.6)	5/3.6	1.4 (0.6 to 3.3)	6.5 (0.3 to 159.3)
50 and older	23/12.7	1.8 (1.2 to 2.7)	22.0 (8.8 to 54.9)	0.3 (0.1 to 0.6)	10/5.1	2.0 (1.1 to 3.6)	25.6 (7.2 to 91.0)	3/2.7	1.1 (0.4 to 3.5)	3.4 (0.0 to 98 057.0)
*P* for trend		<.001	<.001	.001		.02	.06		.34	.14
Chemotherapy^b5^										
No	40/20.9	1.9 (1.4 to 2.6)	7.8 (4.0 to 14.8)	1.0 (referent)	24/12.3	2.0 (1.3 to 2.9)	8.4 (3.7 to 19.1)	16/8.5	1.9 (1.2 to 3.1)	7.2 (2.5 to 20.4)
Yes	58/29.2	2.0 (1.5 to 2.6)	5.6 (3.3 to 9.4)	0.7 (0.4 to 1.4)	40/19.3	2.1 (1.5 to 2.8)	6.7 (3.7 to 12.3)	12/7.6	1.6 (0.9 to 2.8)	3.0 (0.6 to 14.0)
*P* for heterogeneity		.86	.46	.34		.82	.68		.64	.33
Radiotherapy ^b5^										
No	30/16.7	1.8 (1.3 to 2.6)	5.1 (2.3 to 11.4)	1.0 (referent)						
Yes	69/33.4	2.1 (1.6 to 2.6)	7.6 (4.8 to 12.1)	1.4 (0.8 to 2.2)						
*P* for heterogeneity		.52	.37	.21						

Abbreviations: AER = absolute excess risk; CI = confidence interval; E = expected number; O = observed number; RR = relative risk; SIR = standardized incidence ratio.

a It is notable that treatment data were available for 68% of the total cohort.

b Relative risks were derived from 5 different Poisson regression models. For each exposure (factor) a specific Poisson regression model was applied including a different set of confounders. A directed acyclic graph was designed to guide the potential confounding factors. Country was considered as a random effect in each model. b1: No adjustment required (see https://dagitty.net/mHtRRBr55). b2: No adjustment required (see https://dagitty.net/m8u8W5oFt). b3: Relative risks were derived from a Poisson regression including age at diagnosis, and type of childhood cancer (see https://dagitty.net/mVAmNzxtn). b4: Relative risks were derived from a Poisson regression including attained age, type of childhood cancer, decade of childhood cancer, and age at diagnosis (see https://dagitty.net/mRGomGpWk). b5: Relative risks were derived from a Poisson regression including chemotherapy, radiotherapy, type of childhood cancer, and decade of childhood cancer (see https://dagitty.net/mGghodMWs).

### Cumulative incidence

**Figure 1.**
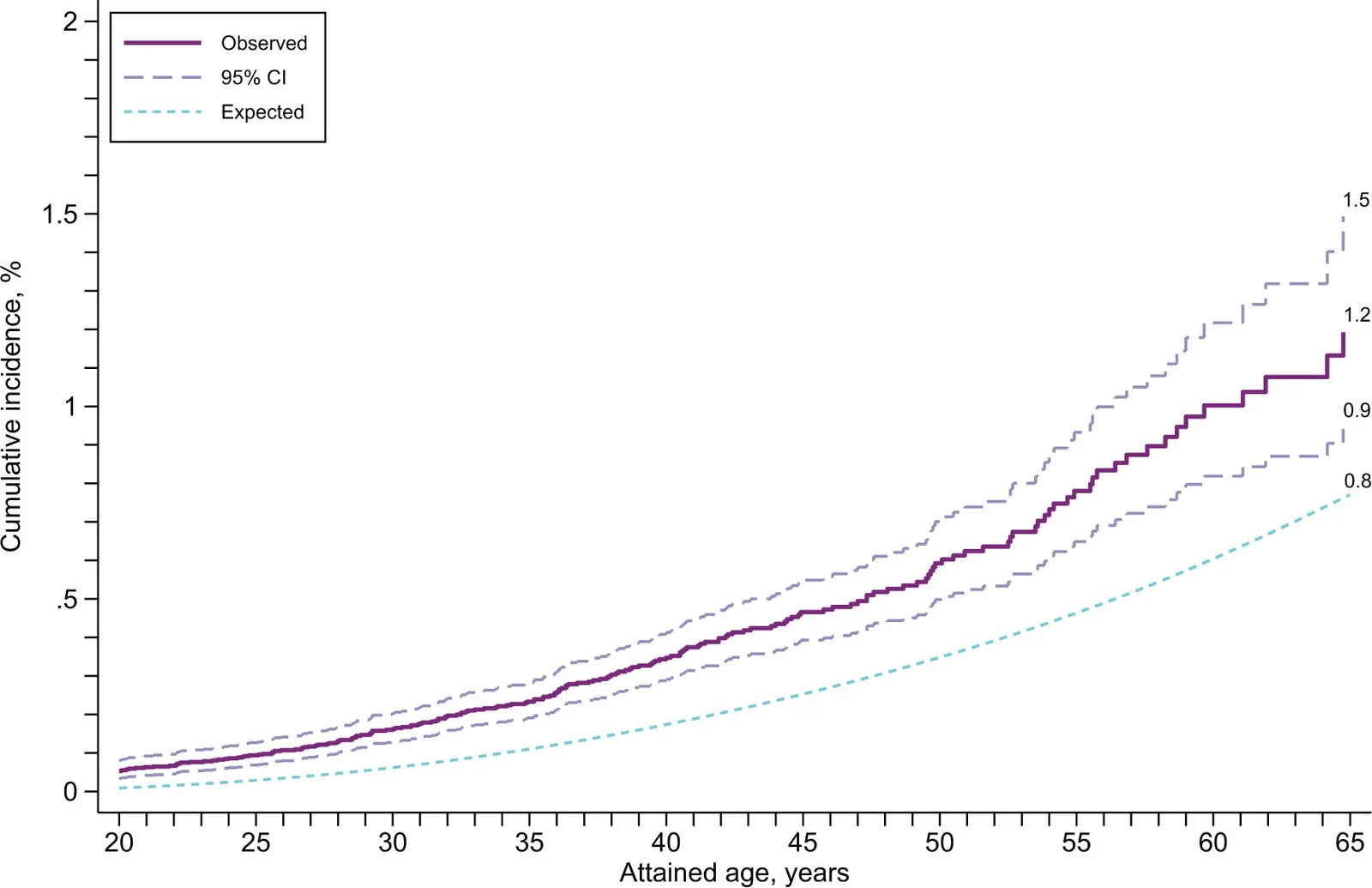
Overall cumulative incidence of subsequent primary melanoma by attained age. Abbreviation: CI = confidence interval.

**Figure 2.**
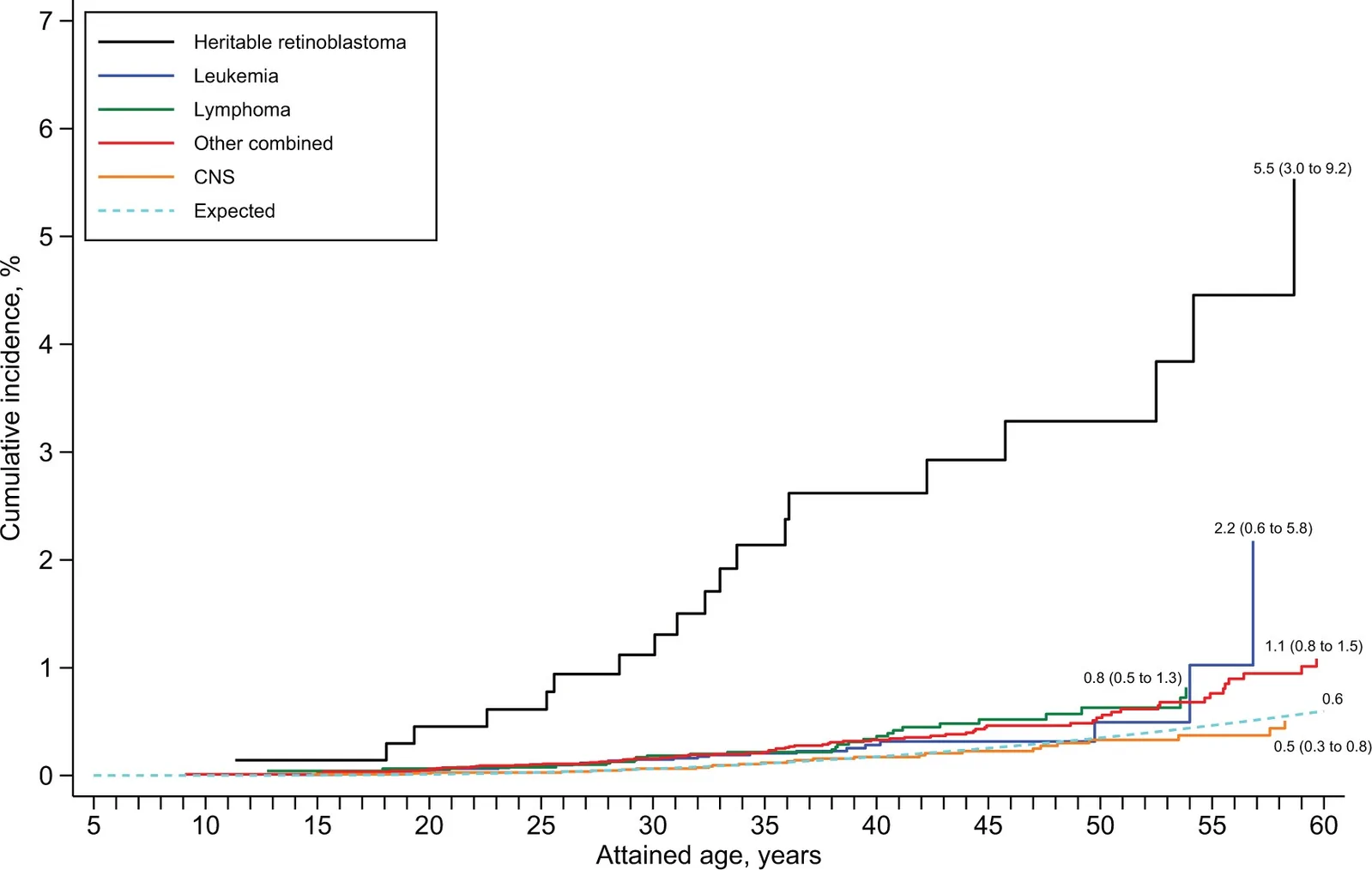
Cumulative incidence of subsequent primary melanoma by attained age and type of childhood cancer. Abbreviation: CNS = central nervous system.

The cumulative incidence of subsequent primary melanoma increased with attained age from 0.1% at age 20 years to 1.2% at age 65 years (Figure 1). Heritable retinoblastoma survivors showed the sharpest increase: 0.5% (95% CI = 0.1% to 1.3%) by age 20 years, 3.3% (95% CI = 1.9% to 5.2%) by age 50 years, and 5.5% (95% CI = 3.0% to 9.2%) by age 60 years. The cumulative incidence reached 2.2% (95% CI = 0.6% to 5.8%) by age 60 years among leukemia survivors and 0.8% (95% CI = 0.5% to 1.3%) by age 55 years among lymphoma survivors. The expected risk in the general population was 0.6% by age 60 years (Figure 2).

**Table:** 

Figure 2. Number at risk and events by attained age group for Figure 2							
Type of childhood cancer		Attained age, y					
		0-19	20-29	30-39	40-49	50-59	60 and older
Heritable retinoblastoma	Events	3	4	7	2	4	1
	At risk	713	568	447	280	152	47
Leukemia	Events	6	11	8	2	2	0
	At risk	15 836	11 084	6482	2374	363	26
Lymphoma	Events	2	9	8	7	2	1
	At risk	7154	8886	5662	2917	1069	291
Other combined	Events	8	17	23	14	13	1
	At risk	19 031	19 463	13 284	7 237	3 066	929
Central nervous system	Events	2	5	9	7	3	1
	At risk	12 800	11 201	7256	4060	1845	551

Values in parentheses denote 95% confidence intervals.The “Other combined” category includes neuroblastoma, Wilms tumor, osteosarcoma, soft tissue sarcoma, germ cell and gonadal neoplasm, nonmelanoma skin cancer, thyroid carcinoma, other and unspecified carcinoma, unspecified lymphoma, nasopharyngeal carcinoma, renal carcinoma, other peripheral nervous cell tumor, other, and unspecified malignant neoplasm. Nonheritable and unknown heritable status retinoblastoma were excluded from analysis.The lymphoma category includes both Hodgkin lymphoma and non-Hodgkin lymphoma.Expected cumulative incidence was similar across childhood cancer types, reaching approximately 0.6% by age 60 years.

### Risk by previous treatment

Survivors treated with radiotherapy had a 70% increased risk compared with those treated without radiotherapy (RR = 1.7, 95% CI = 1.1 to 2.7) (Table 2). This association was strongest in survivors aged 50 years and older (RR = 3.9, 95% CI = 1.1 to 13.9) (Table S3). For prior chemotherapy no statistically significant association was found compared with no chemotherapy (RR = 0.9, 95% CI = 0.5 to 1.6) (Table 2).

## Discussion

In this largest-ever cohort study of childhood cancer survivors, we provide for the first time robust estimates of increased relative and absolute excess risks of subsequent primary melanomas beyond age 50 years and among a wide spectrum of type of childhood cancer survivors. The risk of developing melanoma relative to the general population decreases with attained age but remains increased even beyond age 50 years. Survivors of heritable retinoblastoma are at greatest risk with a 5.5% absolute risk by age 60 years. Additionally, survivors treated with radiotherapy are at increased risk of developing subsequent primary melanoma, even beyond age 50 years compared with survivors treated without radiotherapy.

Few previous studies evaluated subsequent primary melanoma risk by attained age. The largest previous study, conducted in North America, reported elevated standardized incidence ratios and absolute excess risks for melanoma at ages 40-49 years,^11^ consistent with our standardized incidence ratio (SIR = 2.0, 95% CI = 1.4 to 2.7) and absolute excess risk (AER = 16.4, 95% CI = 8.6 to 31.0) at the same ages. To our knowledge, that study is the only 1 to have reported risk estimates beyond age 50 years; however, only 10 melanomas were observed at these ages, resulting in an increased but statistically insignificant standardized incidence ratio (SIR = 1.3, 95% CI = 0.7 to 2.7) and absolute excess risk (AER = 16.6, 95% CI = −15.1 to 79.2). In the present study, with greater statistical power, we identified a downward trend in standardized incidence ratios and an upward trend in absolute excess risks with attained age, with both remaining statistically significantly elevated beyond age 50 years.

Few previous studies reported standardized incidence ratios and/or absolute excess risks of melanoma by type of childhood cancer, and most included few melanomas,^12^^,^^26^ with the exception of retinoblastoma.^27-32^ To our knowledge, standardized incidence ratios have been reported for only 5 cancer types: soft tissue sarcoma (observed melanomas = 2), Hodgkin lymphoma (*n* = 6), leukemia (*n* = 2), and germ cell tumors (*n* = 2), all from the same study,^12^ as well as retinoblastoma (*n* ≤ 40).^27-32^ Our study expands this evidence with 8.0-fold more observations of melanoma for soft-tissue sarcoma, 3.0-fold for Hodgkin lymphoma, 14-fold for leukemia, and 5.0-fold for gonadal and germ cell tumors. The standardized incidence ratios reported in the previous study^12^ were generally consistent with our findings. We ascertained 36 melanomas after retinoblastoma of which 21 were heritable. All previous large-scale cohort studies consistently reported substantially elevated standardized incidence ratios of melanoma following heritable retinoblastoma^27-32^ with our standardized incidence ratio (SIR = 16.5, 95% CI = 10.8 to 25.3) being consistent with all except the standardized incidence ratio reported by Marees et al.,^27^ which was substantially greater (SIR = 50.8, 95% CI = 27.0 to 86.8). Additionally, we report, to our knowledge, for the first time elevated standardized incidence ratios and absolute excess risks for central nervous system tumor, Wilms tumor, neuroblastoma, and non-Hodgkin lymphoma with sufficient melanomas to reliably quantify the risk in these groups. Although heritable retinoblastoma survivors showed substantially greater risks than all other cancer types, the consistently elevated risk across almost all childhood cancer types (standardized incidence ratio approximately 2.0-fold) suggests that melanoma surveillance may warrant consideration for childhood cancer survivors more broadly, not only for heritable retinoblastoma survivors for whom surveillance is already recommended.^33^

Approximately 10% of childhood cancers involve cancer predisposition syndromes,^34^^,^^35^ which may also increase subsequent melanoma risk^36^^,^^37^ independent of prior treatment. Heritable retinoblastoma has been associated with developing subsequent osteosarcoma and soft-tissue sarcoma, as well as melanoma.^38^ The elevated melanoma risk in heritable retinoblastoma survivors is likely driven by germline inactivation of the RB1 tumor suppressor gene, which plays a central role in cell cycle regulation and predisposes to multiple subsequent neoplasm types, including melanoma. In our study, heritable retinoblastoma survivors had 5-times greater risks than nonheritable survivors, although nonheritable retinoblastoma survivors were also at increased risk. However, this finding was based on only 7 melanomas in nonheritable retinoblastoma and should be interpreted cautiously. Genetic testing may not have been routinely performed for all retinoblastoma survivors in our historical cohort, and thus, heritable status may not always definitively have been determined. Consequently, some heritable retinoblastoma survivors may have been misclassified as nonheritable, potentially inflating risks among nonheritable retinoblastoma survivors. The comparable magnitude of risk irrespective of radiotherapy exposure further supports genetic susceptibility as the predominant driver of melanoma risk in heritable retinoblastoma survivors. Notably, heritable retinoblastoma survivors showed similarly elevated standardized incidence ratios regardless of radiotherapy treatment (16.7 with vs 13.6 without radiotherapy; Table 2), although the latter estimate is based on only 2 melanomas.

Other cancer predisposition syndromes associated with melanoma include familial atypical multiple mole melanoma and Li-Fraumeni syndrome (TP53 mutations).^39^^,^^40^ Familial atypical multiple mole melanoma is likely most prevalent among survivors with a first primary melanoma; by excluding individuals with a melanoma as their childhood cancer, the percentage of survivors with familial atypical multiple mole melanoma in our study is likely to be small. Li-Fraumeni syndrome may also be associated with melanoma, but we lacked genetic data to assess the contribution of Li-Fraumeni or other genetic syndromes.

Our finding of a 1.7 increased relative risk among survivors treated with radiotherapy vs those without complements those from Rotz et al.,^11^ who showed a dose–response relationship with 2.0-fold increased risks for body parts receiving cumulative radiation doses exceeding 40 Gy exposure compared with no exposure. Most previous studies focusing on melanoma as a subsequent primary neoplasm could not detect an association between melanoma and treatment with radiotherapy,^10^^,^^12^^,^^14^^,^^41^ although Guerin et al.^41^ found a statistically nonsignificant increased risk for doses exceeding 15 Gy. Likely, the risk is only evident after exposure to high doses of radiation, and most previous studies lacked statistical power to detect an association.

Regarding chemotherapy, we did not find a statistically significant association with the risk of melanoma. Turcotte et al.^42^ did find a 2.0-fold increased risk among survivors treated with chemotherapy only in the Childhood Cancer Survivor Study (CCSS) cohort, and in the most recent CCSS^11^ an increased risk associated with a cyclophosphamide equivalent dose exceeding 20 000 mg/m^2^ and any dose of bleomycin was found. However, we were only able to investigate the risk of melanoma by whether survivors had received any chemotherapy, but not by cumulative doses of drug groups or individual cytotoxic agents.

We demonstrate that survivors remain at increased risk of subsequent primary melanoma throughout all the ages investigated and that the absolute excess risk is greatest above age 50 years. The sixth version of the Long-Term Follow-Up Guidelines for Survivors of Childhood, Adolescent, and Young Adult Cancers from the Children’s Oncology Group recommends periodic evaluations, including annual dermatological examinations and monthly skin self-examinations, as well as sun protection and oncology consultations,^43^ but these recommendations are currently limited to individuals with a history of hematopoietic stem cell transplantation, radiotherapy, experience of any type of skin cancer, or family history of skin cancer. Similarly, the Dutch guideline for follow-up after childhood cancer—more than 5 years after diagnosis updated in 2023—indicates individuals with a history of any type of radiotherapy and hematopoietic stem cell transplantation as people at risk of later melanoma.^44^ Additionally, the 2024 update of PanCare Follow-Up Recommendations for Surveillance of Late Effects suggested self-examination at least every 6 months and a skin examination at least every 2 years after 5 years in individuals with a history of any radiotherapy and or hematopoietic stem cell transplantation.^45^ There are currently no specific recommendations tailored to survivors based on the type of childhood cancer or attained age group. Although treatment-related risk factors have been addressed in existing guidelines, first primary tumor–specific risks have not yet been incorporated. Given that detailed treatment histories are often unavailable, particularly for older survivors in long-term follow-up because of missing or incomplete records, our findings could enhance guidelines by including childhood cancer type and attained age as an additional risk-stratifying factor.

We suggest that guidelines consider incorporating regular dermatological examinations into the long-term follow-up care for these high-risk subgroups to facilitate early detection of melanoma (and nonmelanoma skin cancers). Furthermore, we suggest general melanoma risk reduction strategies, such as regular skin self-examinations, sun protection, and avoidance of intermittent sun exposure, for all survivors.

Our study has several limitations. We lacked information on individual-level melanoma risk factors such as genetic predisposition (eg, CDKN2A mutations), hematopoietic stem cell transplantation, sun exposure, skin phototype, and naevus count. These factors are not routinely captured in population-based cancer registries and would typically affect survivors and the general population similarly, minimizing potential bias. Additionally, we lacked detailed treatment data precluding assessment of risks by cumulative doses of specific chemotherapy agents, cumulative doses of radiation, or specific radiation fields. We could only investigate risks by whether survivors had received radiotherapy but not by the exact anatomical site of the radiation treatment. Although this may have introduced misclassification, it would likely attenuate rather than inflate our risk estimates. Despite these limitations, the association we identified between radiotherapy and melanoma is consistent with findings from Rotz et al.^11^ using more detailed treatment data from the North American CCSS. Furthermore, detailed pathological characteristics of the melanomas, such as Breslow thickness and staging, were not available centrally because of data protection constraints inherent to multicountry studies and could therefore not be investigated.

In conclusion, childhood cancer survivors, particularly heritable retinoblastoma survivors and those who received radiotherapy, remain at risk of melanoma beyond age 50 years. Current guidelines for melanoma risk mainly focus on treatment history. However, our findings suggest that guidelines, particularly long-term follow-up ones, could be enhanced by including childhood cancer type and attained age as additional risk-stratifying factors, particularly when individual treatment histories are not available.

## Supplementary Material

djag094_Supplementary_Data

## Data Availability

Access to anonymized data may be granted under conditions agreed with the relevant (local) legal and research ethics committees and with appropriate data sharing agreements and permissions from each data provider in place. Any data sharing would have to comply with the EU General Data Protection Regulation. The data that support the findings of this study are not publicly available due to privacy and ethical restrictions. Aggregated data in the form of tables may be available on reasonable request.
