# Supplementary material for: Risk of subsequent primary melanoma in 68 002 five-year survivors of childhood and adolescent cancer in Europe: the PanCareSurFup cohort study
Source: J Natl Cancer Inst. 2026 Mar 30;118(9):1670–82. doi: 10.1093/jnci/djag094 (PMC13549720; doi:10.1093/jnci/djag094)
Supplement: Supplementary file 1 [file djag094_supplementary_data.docx]

Supplementary material

Risk of Subsequent Primary Melanoma in 68,002 five-year survivors of childhood and adolescent cancer in Europe: The PanCareSurFup cohort study

Behnoush Shojaie^1^, Jop C Teepen^2^, David L Winter^1^, Mike P Gardner^3^, Melanie Kaiser^4^; Desiree Grabow^4^; Cécile M Ronckers^4,5^; Julianne Byrne^6^; Roderick Skinner^7^; Daniela Alessi^8^; Hama Baqaeen^1^; Rodrigue S Allodji^9,10^; Helena JH van der Pal^2^; Momcilo Jankovic^11^; Francesca Bagnasco^12^; Eva Steliarova-Foucher^13^; Ben Spycher^14^; Grit Sommer^14^; Edit Bárdi^15,16^; Thorgerdur Gudmundsdottir^17,18^; Milena M Maule^19^; Gisela Michel^20^; Monica Muraca^21^; Maria W Gunnes^22,23^; Line Kenborg^24^; Henrik Hjalgrim^25^; Carlotta Sacerdote^19,26^; Zsuzsanna Jakab^27^; Riccardo Haupt^21^; Thomas Wiebe^28^; Monica Terenziani^29^; Lorna Zadravec Zaletel^30,31^; Leontien CM Kremer^2,32^; Claudia E Kuehni^14,33^; Päivi M Lähteenmäki^34^; Florent de Vathaire^9,10^; Lars Hjorth^35^; Michael M Hawkins^1^; Raoul C Reulen^1^

1. Centre for Childhood Cancer Survivor Studies, Department of Applied Health Sciences, University of Birmingham, Birmingham, UK
2. Princess Máxima Centre for Paediatric Oncology, Utrecht, The Netherlands
3. Department of Applied Health Sciences, University of Birmingham, Birmingham, UK
4. German Childhood Cancer Registry, Division of Childhood Cancer Epidemiology, Institute of Medical Biostatistics, Epidemiology and Informatics (IMBEI), Johannes-Gutenberg University Mainz, Mainz, Germany
5. Division of CAYA Cancer Survivorship Research. German Cancer Research Centre, Heidelberg, Germany,
6. Retired from the Boyne Research Institute, Drogheda, Ireland
7. Great North Children's Hospital, Newcastle upon Tyne Hospitals NHS Foundation Trust and Newcastle University Centre for Cancer, Newcastle University, Newcastle upon Tyne, UK
8. Childhood Cancer Registry of Piedmont, Cancer Epidemiology Unit, Department of Medical Sciences, University of Turin and CPO-Piemonte, AOU Città della Salute e della Scienza di Torino, Turin, Italy
9. Radiation Epidemiology Team, Centre for Research in Epidemiology and Population Health, INSERM U1018, University Paris Saclay, Gustave Roussy, Villejuif, France
10. Radiation Epidemiology (EpiRad) Team, Gustave Roussy, Villejuif, France
11. Paediatric Clinic, Maria Letizia Verga Foundation, IRCCS San Gerardo, Monza, Italy
12. Epidemiology and Biostatistics Unit, IRCCS Istituto Giannina Gaslini, Genova, Italy
13. Cancer Surveillance Branch, International Agency for Research on Cancer (IARC), Lyon, France
14. Childhood Cancer Research Group, Institute of Social and Preventive Medicine, University of Bern, Bern, Switzerland
15. St Anna Children’s Hospital, Vienna, Austria
16. Department of Paediatrics and Adolescent Medicine, Johannes Kepler University Linz, Kepler University Hospital, Linz, Austria
17. Children´s Hospital, Landspitali University Hospital, Reykjavik, Iceland
18. Childhood Cancer Research Group, Danish Cancer Institute, Copenhagen, Denmark
19. Cancer Epidemiology Unit, Department of Medical Sciences, University of Turin and AOU Citta della Salute e della Scienza, CPO-Piemonte, Turin, Italy
20. Faculty of Health Sciences and Medicine, University of Lucerne, Lucerne, Switzerland
21. DOPO clinic, Division of Haematology/Oncology, IRCCS Istituto Giannina Gaslini, Genova, Italy
22. Department of Registration, Cancer Registry of Norway, Oslo, Norway
23. National Advisory Unit on solid tumours in childhood, Division of Paediatric and Adolescent Medicine, Oslo University Hospital Rikshospitalet, Oslo, Norway
24. Childhood Cancer Research Group, Danish Cancer Institute, Copenhagen, Denmark
25. Haematology, Danish Cancer Institute, Copenhagen, Denmark
26. Centre for Cancer Epidemiology and Prevention (CPO Piemonte), Turin, Italy
27. Hungarian Childhood Cancer Registry, Hungarian Paediatric Oncology Network, Department of Paediatrics, Semmelweis University, Budapest, Hungary
28. Department of Clinical Sciences Lund, Paediatrics, Skane University Hospital, Lund University, Lund, Sweden
29. Pediatric Oncology Unit, Fondazione IRCCS Istituto Nazionale dei Tumori, Milan, Italy
30. Division of Radiotherapy, Institute of Oncology, Ljubljana, Slovenia
31. Faculty of Medicine, University of Ljubljana, Ljubljana, Slovenia
32. Emma Children’s Hospital, Amsterdam UMC, Amsterdam, The Netherlands
33. Division of Paediatric Hematology/Oncology, Department of Paediatrics, University Children's Hospital of Bern, Bern, Switzerland
34. Turku University and Turku University Hospital, Department of Paediatrics and Adolescent Medicine, Turku, Finland
35. Lund University, Skane University Hospital, Department of Clinical Sciences Lund, Paediatrics, Lund, Sweden

| **Table S1.** Characteristics of five-year survivor cohorts contributing to the European PanCareSurFup cohort, by country | | | | | | | |
| --- | --- | --- | --- | --- | --- | --- | --- |
| **Cohort** | **No. of 5-year Survivors** | **Period of Diagnosis** | **Study Design** | **Age at childhood cancer (yrs)** | **Study exit date^f^** | **% Loss to Follow-Up** | **Methods of SPM Ascertainment** |
| France | 3,130 | 1946-1986 | Hospital-based | 0-18 | Sep-14 | — | Multiple methods ^a^ |
| Hungary | 4,869 | 1971-2008 | Population-based | 0-19 | Dec-14 | — | Multiple methods ^b^ |
| Italy^g^ | 8,815 | 1960-2007 | Population-based | 0-19 | May-10 | 2 | Multiple methods ^c^ |
| Netherlands | 6,022 | 1963-2001 | Population-based | 0-17 | Dec-12 | 6 | Multiple methods ^d^ |
| Denmark | 4,615 | 1943-1998 | Population-based | 0-19 | Dec-03 | 5 | Population based |
| Sweden | 7,418 | 1958-1998 | Population-based | 0-19 | Dec-03 | 2 | Population based |
| Norway | 3,537 | 1953-1997 | Population-based | 0-19 | Dec-02 | 3 | Population based |
| Finland | 6,056 | 1953-2006 | Population-based | 0-19 | Dec-11 | 5 | Population based |
| Iceland | 261 | 1955-1998 | Population-based | 0-19 | Dec-03 | 5 | Population based |
| Slovenia | 1,221 | 1960-2002 | Population-based | 0-16 | Jul-14 | 5 | Population based |
| Switzerland | 4,239 | 1964-2005 | Population-based | 0-19 | Dec-13 | — | Multiple methods ^e^ |
| United Kingdom | 17,819 | 1940-1991 | Population-based | 0-15 | Sep-16 | 5 | Population based |
| Total | 68,002 | 1940-2008 |  | 0-19 |  |  |  |
| Abbreviations: SPM=subsequent primary melanoma  a: Multiple methods include long-term follow-up clinics, questionnaires to survivors, national mortality records and health insurance registries.  b: Multiple methods include long-term follow-up clinics, questionnaires to survivors and medical records/hospital data.  c: Multiple methods include medical records/ hospital data, national mortality records.  d: Multiple methods include population-based cancer registries, long-term follow-up clinics and medical records/hospital data.  e: Multiple methods include population-based cancer registries, questionnaires to survivors, long-term follow-up clinics, and national mortality records.  f: For France, Hungary, Italy and Switzerland this is not the definitive study end date, but the last known date of exit, as individuals were on active follow-up so there was not one fixed study end date.  g: Italy consisted of one population-based cohort and one treatment centre-based cohort  — Could not calculate as there was no definitive study end date | | | | | | | |

| **Table S2**. Median latency to first melanoma and age at melanoma development, by childhood cancer type | | |
| --- | --- | --- |
| **Type of childhood cancer** | **Median latency**  **(IQR) (years)** | **Median age**  **development**  **(IQR) (years)** |
| All survivors | 26.5 (19.2–36.5) | 35.2 (26.9–44.0) |
| leukaemia | 20.6 (15.0–28.0) | 27.7 (20.9–34.3) |
| Hodgkin lymphoma | 24.8 (13.0–25.6) | 38.8 (29.2–41.2) |
| non-Hodgkin lymphoma | 23.3 (13.5–27.3) | 31.5 (28.6–41.1) |
| CNS tumour | 28.7 (22.9–39.7) | 36.3 (29.2–47.3) |
| neuroblastoma | 34.4 (21.5–39.5) | 35.3 (22.0–40.6) |
| retinoblastoma | 33.9 (26.2–44.5) | 36.0 (27.6–46.2) |
| Wilms tumour | 31.0 (25.2–36.9) | 31.6 (27.2–41.2) |
| bone sarcoma | 24.5 (17.1–28.8) | 38.4 (31.0–42.8) |
| soft-tissue sarcoma | 25.8 (17.2–33.5) | 36.3 (25.1–50.5) |
| germ cell and gonadal neoplasm | 23.9 (16.5–39.1) | 37.6 (32.8–49.7) |
| others ^a^ | 26.9 (15.8–36.5) | 37.8 (24.5–44.8) |
| Abbreviations: IQR= Interquartile Range; CNS=central nervous system.  a: Included: non-melanoma skin cancer, thyroid carcinomas, other and unspecified carcinomas, unspecified lymphomas, nasopharyngeal carcinoma, renal carcinoma, other peripheral nervous cell tumour, other and unspecified malignant neoplasm. | | |

| **Table S3**. Relative Risk of SPM for radiotherapy stratified by attained age | | | | | | |
| --- | --- | --- | --- | --- | --- | --- |
| **Radiotherapy** | | **Attained age (years)** | | | | |
|  |  | **5-19** | **20-29** | **30-39** | **40-49** | **over 50** |
| RR (95% CI) | No | 1 (ref) | 1 (ref) | 1 (ref) | 1 (ref) | 1 (ref) |
|  | Yes | 3.9 (0.9, 15.8) | 3.0 (1.0, 9.5) | 0.6 (0.3, 1.3) | 1.4 (0.5, 3.5) | 3.9 (1.1, 13.9) |
| *P-value* | | *0.062* | *0.054* | *0.230* | *0.534* | *0.034* |
| Abbreviations: CI=confidence interval; RR=relative risk  The Poisson regression model stratified by attained age including radiotherapy, chemotherapy, type of childhood cancer, and decade of childhood cancer. Country was included as a random effect in the Poisson regression model | | | | | | |
